# A Department of Public Health in the U. S. Government

**Published:** 1897-03

**Authors:** I. N. Love


### A Department of Public Health in the U. S. Government.

"Surely, if the health of an individual is important, the health of nations is much more important, and it seems strange that it should be necessary to present any arguments to secure a proper recognition of the importance of public health by the government. The legal necessities of the government are recognized by the provision which makes the Attorney-General a member of the President's Cabinet. The finances are represented by the Secretary of the Treasury. Our relations with foreign nations are properly met by the Secretary of State, and his department subordinates. So, too, the needs of the army and navy are met by their respective representatives in the Cabinet. Even the rights and necessities of Lo, the poor Indian, are provided for by the Secretary of the Interior. Recently the great agricultural interests of America have been dignified by the creation of a Cabinet officer with the title of Secretary of Agriculture. That there was not long ago similar recognition given to the sanitary and medical necessities of the country by the creation of a department of public health, giving its chief a place in the Cabinet of the President, is the fault of the medical profession. All that is necessary to secure this great need is that the profession take united action.

No class in a community has really a greater influence than the doctors if they but act in harmony. The medical man goes into every home in the land, and is in a position to wield a mighty influence for the good of the people at large and in behalf of his own profession. It seems to me that the best way to create this special department would be to build upon the foundations that have been already erected in the form of the quarantine and sanitary department of the United States Marine Hospital Service. As is well known, this service has had, for many years, complete charge of the quarantine ports, and been in close communication, through the consular service and its representatives in foreign ports, with the quarantine service of the world. It would not be a difficult task for the Marine Hospital arm of the government to be taken away from the Treasury Department and strengthened and dignified in good form, and crystalized in the direction of an independent branch of the government service, known as the Public Health Department. The efficiency of its work would be the better accomplished, its dignity the more completely emphasized by making its chief a member of the Cabinet of the President of the United States. Let us hope that the powers that be, may be ere long impressed with the importance of this suggestion. If fully informed as to the great money value, aside from the humanitarian view, of the prevention of disease in the nation at large, the suggestion would soon become an accomplished fact.

"If the editorial guild medical would write and demand, through the columns they control, action upon the part of the profession, they could wield a mighty influence. Let us determine to do our part in the work."--*I. N. Love, in the American Journalist.*

